# Long-term cardiovascular risk reduction after gastric cancer surgery: a nationwide cohort study

**DOI:** 10.1097/JS9.0000000000001404

**Published:** 2024-03-27

**Authors:** Yeongkeun Kwon, Dohyang Kim, Sangwoo Kim, Jane Ha, Jinseub Hwang, Sungsoo Park, Jin-Won Kwon

**Affiliations:** a Division of Foregut Surgery, Korea University College of Medicine; b Center for Obesity and Metabolic Diseases, Korea University Anam Hospital, Seoul; c Department of Statistics, Daegu University, Gyeongbuk; d Department of Medicine, Korea University College of Medicine, Seoul; e BK21 FOUR Community-Based Intelligent Novel Drug Discovery Education Unit, College of Pharmacy and Research Institute of Pharmaceutical Sciences, Kyungpook National University, Daegu, South Korea; f Clinical and Translational Epidemiology Unit, Massachusetts General Hospital, Boston, Massachusetts, United States

**Keywords:** cardiovascular risk, endoscopic resection, gastrectomy, gastric cancer

## Abstract

**Background:**

Gastrectomy for gastric cancer is associated with postoperative changes in cardiovascular risk factors, however, the impact of gastrectomy on cardiovascular events remains unclear. The authors assessed the incidence of cardiovascular events between patients undergoing gastrectomy or endoscopic resection for gastric cancer, and the general population.

**Materials and methods:**

This retrospective nationwide cohort study included patients with gastric cancer undergoing gastrectomy (*n*=37 698), endoscopic resection (*n*=2773), and matched control population (*n*=161 887) between 2004 and 2013. The authors included patients without a history of cancer other than gastric cancer, myocardial infarction, or ischemic stroke. The primary outcome was the incidence of major adverse cardiovascular events (MACE) such as acute myocardial infarction, revascularization, or acute ischemic stroke, in patients with gastric cancer.

**Results:**

Among patients who underwent gastrectomy for gastric cancer, 2.9% (4.69 per 1000 person-years) developed novel MACE within the 1-year follow-up period. The gastrectomy group demonstrated a significantly decreased risk for MACE than the control population [hazard ratio (HR), 0.65; 95% CI: 0.61–0.69; *P*<0.001). Among the patients undergoing endoscopic resection for gastric cancer, 5.4% (8.21 per 1000 person-years) developed novel MACE within the 7-year follow-up period. The risk for MACE in the endoscopic resection group was not significantly different from the control population.

**Conclusion:**

Patients with gastric cancer who have undergone gastrectomy exhibit a reduced risk of cardiovascular diseases in comparison to the general population. In contrast, the risk for cardiovascular diseases in patients with gastric cancer who underwent endoscopic resection did not demonstrate a significant difference in cardiovascular risk in comparison to the general population.

## Introduction

Highlights

Patients with gastric cancer who have undergone gastrectomy have a reduced risk of cardiovascular disease compared to the general population.
In contrast, patients with gastric cancer who underwent endoscopic resection did not show a significant difference in cardiovascular risk compared with the general population after treatment.
Our results, in addition to their public health importance for the management of cardiovascular risk in patients with gastric cancer, will help patients and healthcare providers make more informed decisions regarding gastric cancer surgery.
In addition, our results provide important research hypotheses regarding the metabolic effects of gastric cancer surgery, such as a reduction in cardiovascular risk.

Patients with cancer have a 20-fold increased risk of cardiovascular disease, which is the leading cause of noncancer mortality among patients with cancer^1^. The increase in cardiovascular disease in patients with cancer can be attributed to both the cancer itself and the cancer treatment modality, which can be damaging to cardiovascular health^2^. This is of particular concern in gastric cancer, for which gastrectomy is the gold standard treatment. Given that gastrectomy is associated with postoperative weight loss and changes in the natural progression of comorbidities such as type 2 diabetes, hypertension, and dyslipidemia, both commonly known risk factors for cardiovascular disease^3–6^. Considering that the management of cardiovascular disease in patients with gastric cancer is closely related to both the survival rate and quality of life of the patient^2^, assessing the risk of cardiovascular disease after gastrectomy is critical.

Depending on the lesion stage, the primary treatment modalities for gastric cancer are gastrectomy and endoscopic resection, both of which alter cardiovascular risk factors differently. In addition to lesion removal, gastric cancer surgery alters metabolism and glucose homeostasis. Owing to its weight reduction and accompanying metabolic effects, gastrectomy for gastric cancer significantly increases the likelihood of type 2 diabetes remission and decreases the likelihood of type 2 diabetes relapse^7,8^. Previous studies have confirmed the benefits of gastric cancer surgery on blood pressure and dyslipidemia management^4,9,10^. Conversely, endoscopic resection is less anatomically and physiologically invasive than gastrectomy, thus allowing for a compatible contrast group for assessing cardiovascular outcomes in patients with gastric cancer who have undergone surgery.

This study aimed to compare the incidence of cardiovascular events between patients undergoing gastrectomy or endoscopic resection for gastric cancer and the general population. We used a nationwide population-based cohort in South Korea, the country with the world’s highest rate of gastric cancer survival^11^, which implies that this is the optimal patient population for studying comorbidities in patients with long-term survival following gastric cancer.

## Methods

### Data source and study population

The National Health Insurance Service (NHIS) is a single insurer managed by the Korean government. The NHIS manages a mandatory universal insurance system that covers the entire Korean population (97% through the health insurance system and 3% through medical aid). Therefore, the NHIS maintains representative population-based cohort data, including a claims database containing medical information on the entire Korean population-based on insurance claims (e.g. patient demographics, disease diagnoses, medical treatments, and procedures). The NHIS database has been extensively described in a previous study^12^.

The NHIS provides biannual health checkups for all insured Koreans aged ≥40 years. Among patients who underwent gastrectomy or endoscopic resection for cancer between 2004 and 2013, 59 358 who underwent health checkups within 2 years pregastric cancer treatments were identified using the International Classification of Diseases, 10th Revision, Clinical Modification (ICD-10-CM) codes for total gastrectomy (Q2533–Q2537, QA533, QA535, and QA536) or subtotal gastrectomy (Q0251–Q0259, Q2594, Q2595, Q2597, Q2598, QA595, QA597, and QA598), endoscopic resection (Q2052, Q7652, QX701), and gastric cancer (C16) (Supplementary Fig. 1, Supplemental Digital Content 1, http://links.lww.com/JS9/C305). Patients with a history of cancer, including gastric cancer (*n*=14 010; ICD-10-CM codes C, except C16), within 2 years prior to gastrectomy or endoscopic resection were excluded. To avoid the potential effects of reverse causality, patients with a history of myocardial infarction or stroke (*n*=2891; ICD-10-CM codes I21, I22, and I63) within 2 years prior to gastrectomy or endoscopic resection were excluded. Patients who developed myocardial infarction or stroke within 2 years of gastrectomy or endoscopic resection (*n*=579) were excluded. After excluding patients with missing variables (*n*=1627), 40 471 patients were enrolled and evaluated for MACE development after gastrectomy or endoscopic resection.

To compare the cardiovascular risk in patients with gastric cancer with that in the general population, the control group was selected as follows: first, among the patients who underwent health checkups between 2004 and 2013, those without a history of cancer, myocardial infarction, or stroke were screened. After excluding patients who developed cancer between 2002 and 2020, the control group was selected by performing 1:4 matching based on age and sex with the gastric cancer patient group. This study was approved by the Institutional Review Board (No. 2019AN0156), which waived the requirement for informed consent because the customized database was released after de-identification and anonymization. This retrospective study has been reported in line with the strengthening the reporting of cohort, cross-sectional, and case–control studies in surgery (STROCSS) criteria^13^ (Supplemental Digital Content 2, http://links.lww.com/JS9/C306).

### Primary outcome

The primary outcome was MACE (a composite of the first occurrence of acute myocardial infarction, revascularization, or acute ischemic stroke). Acute myocardial infarction was defined using the ICD-10-CM codes I21 and I22 during admission. Revascularization was defined based on a claim history for cardiovascular revascularization procedures. Acute ischemic stroke was defined by ICD-10-CM code I63 during admission and a claim history for brain MRI or computed tomography. Patients were followed up until the date of MACE or for seven years after gastric cancer treatments.

### Clinical variables

A standardized self-report questionnaire was used to obtain demographic and lifestyle data. Smoking status was categorized as never smoked, former smoker, or current smoker. Individuals who consumed ≥30 and <30 g of alcohol per day were considered heavy and mild drinkers, respectively. Exercise was recorded as days of moderate-to-vigorous physical activity for more than 20–30 min per week (none, one–four times per week, ≥ five times per week). Low-income status was defined as the bottom 30% of the study population in terms of income level. Fasting plasma glucose (FPG) levels were measured after an overnight fast. Blood pressure was measured after at least 10 min of rest in the sitting position and repeated if the initial measurement was >120/80 mmHg. BMI was calculated as weight in kilograms divided by the square of height in meters, and obesity was defined as BMI ≥25 kg/m^2^ according to the Asia-Pacific criteria of the WHO guidelines^14^. The hospitals in which these health examinations were performed were certified by the NHIS and subjected to regular quality control.

Baseline comorbidities (hypertension, diabetes, dyslipidemia, peripheral arterial disease, heart failure, atrial fibrillation, transient ischemic attack, and chronic kidney disease) were defined using a combination of ICD-10-CM diagnoses, prescription codes, and laboratory results. Cardiovascular medications (ACE inhibitors, angiotensin receptor blockers, antiplatelets, β-blockers, calcium-channel blockers, diuretics, and statins) at baseline were obtained using prescription codes. Detailed definitions of comorbidities are presented in Supplementary Table 1 (Supplemental Digital Content 1, http://links.lww.com/JS9/C305).

### Statistical analysis

Summary data are presented as frequencies with percentages for categorical variables and means with SDs for continuous variables. Gastrectomy or endoscopic resection and control populations were compared using the *χ*^2^ test for categorical variables and the Student’s *t*-test for continuous variables. The incidence rate of MACE was calculated by dividing the number of incident MACE cases by the total follow-up duration (person-years), and Kaplan–Meier curves with log-rank *χ*^2^ were used to compare MACE among the three groups: gastrectomy, endoscopic resection, and the control population. Hazard ratios (HRs) and 95% CIs were estimated using multivariate Cox regression analyses for the three study groups. The Cox model was adjusted for age, sex, BMI, systolic blood pressure, FPG, total cholesterol, smoking status, alcohol consumption, exercise, income status, comorbidities (hypertension, diabetes, dyslipidemia, peripheral arterial disease, heart failure, atrial fibrillation, transient ischemic attack, chronic kidney disease), cardiovascular medications (ACE inhibitors, angiotensin receptor blockers, antiplatelets, β-blockers, calcium-channel blockers, diuretics, statins), and baseline year. Given the competing risks of MACE and mortality in patients undergoing gastric cancer treatments, a competing risk regression model was considered using the Fine and Gray method^15^. To evaluate whether the effects of gastrectomy or endoscopic resection varied across subgroups, we tested the interaction between group assignments and risk factor categories.

Moreover, we investigated the effects of potentially unmeasured confounders. We developed covariate-adjusted Cox models to estimate the HRs for gastrectomy and endoscopic resection compared with the control population. Furthermore, we analyzed whether the observed differences in the rate of MACE, MI, stroke, or revascularization could be fully explained by an unmeasured confounder^16^, and calculated the minimum strength of the confounder-exposure and confounder-outcome associations on the risk ratio scale to fully explain non-null associations (E-values)^17^. The level of statistical significance was set at *P*<0.05. All analyses were two-tailed and performed using SAS software version 9.3 (SAS Institute Inc.).

## Results

### Baseline characteristics

The mean age of the patients in the gastrectomy, endoscopy resection, and control groups was 60.9 years (SD, 11.2), 63.9 years (SD, 9.9), and 61.1 years (SD, 11.1), respectively, with a significant difference between groups (*P*=0.001 for gastrectomy vs. control, *P*<0.001 for endoscopic resection vs. control) (Table 1). The percentages of women in the gastrectomy, endoscopic resection, and control groups were 26.4, 24.3, and 26.3%, respectively. The average BMI in the gastrectomy and endoscopic resection groups and control population was 23.8 kg/m² (SD, 3.0), 24.0 kg/m² (SD 2.9), and 23.9 kg/m² (SD, 2.9), respectively, with a significant difference between gastrectomy vs. control (*P*<0.001). The gastrectomy and endoscopic resection groups were more likely to have fewer nonsmokers (*P*<0.001) and nondrinkers (*P*<0.001). Significant differences were observed in the proportion of comorbidities such as hypertension (*P*<0.001), diabetes (*P*<0.001), dyslipidemia (*P*<0.001), peripheral arterial disease (*P*<0.001 for gastrectomy, *P*=0.008 for endoscopic resection), and atrial fibrillation (*P*<0.001 for gastrectomy, *P*=0.002 for endoscopic resection) between the gastrectomy or endoscopic resection groups and the control population. Significant differences were observed in the proportion of patients taking cardiovascular medications at baseline.

**Table 1:** Baseline characteristics.

	Patients with gastric cancer				
Variables	Gastrectomy (*n*=37 698)	Endoscopic resection (*n*=2773)	Control (*n*=161 887)	P (G vs. C)	P (E vs. C)
Age, years, mean (SD)	60.9 (11.2)	63.9 (9.9)	61.1 (11.1)	0.001	<0.001
Female sex, *n* (%)	9950 (26.4)	673 (24.3)	42495 (26.3)	0.567	0.019
BMI, kg/m^2^, mean (SD)	23.8 (3.0)	24.0 (2.9)	23.9 (2.9)	<0.001	0.765
>18.5, *n* (%)	1155 (3.1)	81 (2.9)	4228 (2.6)	<0.001	0.330
18.5–23, *n* (%)	13838 (36.7)	918 (33.1)	56019 (34.6)		
23–25, *n* (%)	10200 (27.1)	780 (28.1)	44947 (27.8)		
<25, *n* (%)	12505 (33.2)	994 (35.9)	56693 (35.0)		
Systolic blood pressure, mmHg, mean (SD)	126.8 (16.4)	128.5 (16.4)	127.7 (16.9)	<0.001	0.007
Diastolic blood pressure, mmHg, mean (SD)	78.3 (10.5)	79.0 (10.5)	79.1 (10.8)	<0.001	0.687
Fasting plasma glucose, mg/dl, mean (SD)	101.7 (28.4)	102.5 (36.5)	100.9 (30.5)	<0.001	0.023
Total cholesterol, mg/dl, mean (SD)	195.3 (41.5)	195.8 (38.7)	197.9 (42.7)	<0.001	0.005
Smoking status					
Never, *n* (%)	19 613 (52.0)	1532 (55.3)	94 049 (58.1)	<0.001	<0.001
Former, *n* (%)	6900 (18.3)	568 (20.5)	27 675 (17.1)		
Current, *n* (%)	11 185 (29.7)	673 (24.3)	40 163 (24.8)		
Alcohol consumption					
Nonuser, *n* (%)	21 540 (57.1)	1576 (56.8)	92 843 (57.4)	<0.001	<0.001
Mild drinker, *n* (%)	10 741 (28.5)	737 (26.6)	48 880 (30.2)		
Heavy drinker, *n* (%)	5417 (14.4)	460 (16.6)	20 164 (12.5)		
Regular exercise					
None, *n* (%)	15 299 (40.6)	1226 (44.2)	68 094 (42.1)	<0.001	0.002
1–4 per week, *n* (%)	11 611 (30.8)	828 (29.9)	53 544 (33.1)		
≥ 5 per week, *n* (%)	10 788 (28.6)	719 (25.9)	40 249 (24.9)		
Low-income, n (%)	5303 (14.1)	374 (13.5)	23 671 (14.6)	0.006	0.093
Comorbidities					
Hypertension, *n* (%)	13 914 (36.9)	1199 (43.2)	57 688 (35.6)	<0.001	<0.001
Diabetes, *n* (%)	8865 (23.5)	728 (26.3)	30 085 (18.6)	<0.001	<0.001
Dyslipidemia, *n* (%)	11 779 (31.3)	919 (33.1)	38 419 (23.7)	<0.001	<0.001
Peripheral arterial disease, *n* (%)	3749 (9.9)	295 (10.6)	14 848 (9.2)	<0.001	0.008
Heart failure, *n* (%)	775 (2.1)	56 (2.0)	2776 (1.7)	<0.001	0.221
Atrial fibrillation, *n* (%)	571 (1.5)	51 (1.8)	1942 (1.2)	<0.001	0.002
Transient ischemic attack, *n* (%)	636 (1.7)	61 (2.2)	2615 (1.6)	0.322	0.016
Chronic kidney disease, *n* (%)	372 (1.0)	25 (0.9)	1030 (0.6)	<0.001	0.083
Cardiovascular medications					
ACE inhibitors, *n* (%)	1963 (5.2)	195 (7.0)	8737 (5.4)	0.141	<0.001
Angiotensin receptor blockers, *n* (%)	6109 (16.2)	477 (17.2)	23 884 (14.8)	<0.001	<0.001
Antiplatelets, *n* (%)	6115 (16.2)	486 (17.5)	24 493 (15.1)	<0.001	0.001
β-blockers, *n* (%)	3981 (10.6)	338 (12.2)	17 822 (11.0)	0.012	0.049
Calcium-channel blockers, *n* (%)	8132 (21.6)	722 (26.0)	34 399 (21.3)	0.168	<0.001
Diuretics, *n* (%)	3486 (9.3)	274 (9.9)	14 025 (8.7)	<0.001	0.024
Statins, *n* (%)	5211 (13.8)	411 (14.8)	21 160 (13.1)	<0.001	0.007

ACE, angiotensin-converting enzyme; E, endoscopic resection; G, gastrectomy.

### Cardiovascular risk in patients undergoing gastrectomy for gastric cancer

**Figure 1:**
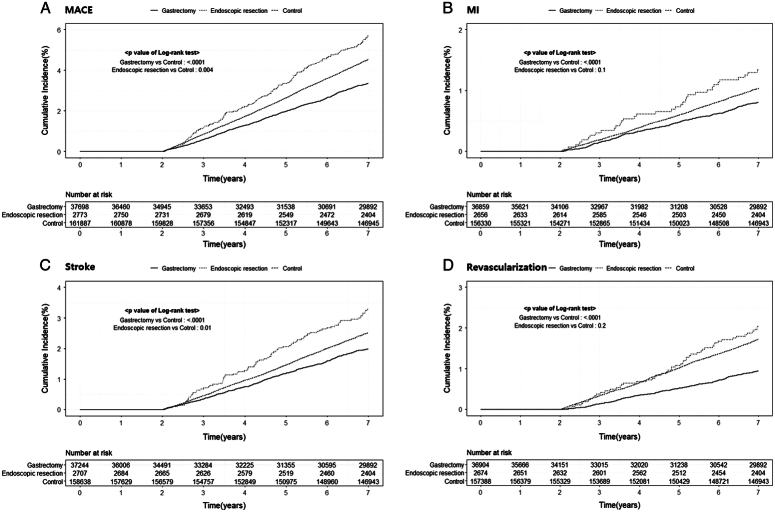
Kaplan–Meier estimates of cardiovascular events in patients undergoing gastrectomy and endoscopic resection for gastric cancer compared to those of control population.

**Figure 2:**
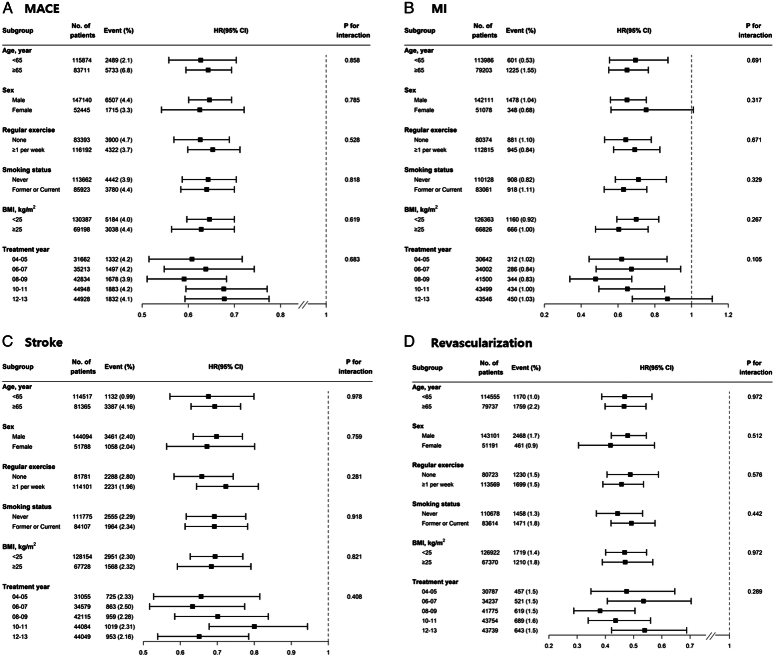
Subgroup analyses according to baseline characteristics (gastrectomy vs. control population); (A) MACE, (B) MI, (C) stroke, (D) revascularization. MACE, major adverse cardiovascular event; MI, myocardial infarction.

Among patients who underwent gastrectomy for gastric cancer, 2.9% (4.69 per 1000 person-years) developed novel MACE during the 7 years follow-up (Table 2). In the Kaplan–Meier curve, significantly decreased risks of MACE (*P*<0.001), MI (*P*<0.001), stroke (*P*<0.001), and revascularization (*P*<0.001) were identified (Fig. 1). The gastrectomy group had a significantly decreased risk of MACE than those in the control group (HR, 0.65; 95% CI: 0.61–0.69; *P*<0.001), MI (HR, 0.67; 95% CI: 0.59–0.76; *P*<0.001), stroke (HR, 0.70; 95% CI: 0.64–0.76; *P*<0.001), and revascularization (HR, 0.47; 95% CI: 0.42–0.53; *P*<0.001). In the subgroup analyses, no significant interactions were identified between the study groups and baseline variables (Fig. 2).

**Table 2:** Cardiovascular risk in patients undergoing gastrectomy or endoscopic resection for gastric cancer compared to control population.

	Event, *n* (%)	Person-years	Incidence, per 1000 person-years	Adjusted HR (95% CI)	*P*
Gastrectomy group vs. control					
MACE	1095 (2.9)	233533	4.69	0.65 (0.61–0.69)	<0.001
MI	256 (0.7)	229752	1.11	0.67 (0.59–0.76)	<0.001
Stroke	641 (1.7)	231476	2.77	0.70 (0.64–0.76)	<0.001
Revascularization	301 (0.8)	230002	1.31	0.47 (0.42–0.53)	<0.001
Endoscopic resection vs. control					
MACE	151 (5.4)	18388	8.21	1.07 (0.91–1.26)	0.405
MI	34 (1.3)	17860	1.90	1.10 (0.78–1.54)	0.597
Stroke	85 (3.1)	18087	4.70	1.10 (0.88–1.37)	0.397
Revascularization	52 (1.9)	17951	2.90	1.03 (0.78–1.36)	0.830

HR, hazard ratio; MACE, major adverse cardiovascular event; MI, myocardial infarction.

HRs (95% CIs) were estimated using multivariable Cox regression and competing risk regression model.

### Cardiovascular risk in patients undergoing endoscopic resection for gastric cancer

Among the patients undergoing endoscopic resection for gastric cancer, 5.4% (8.21 per 1000 person-years) developed novel MACE during the 7-year follow-up (Table 2). Significant differences in the risks of MACE (*P*=0.004) and stroke (*P*=0.010) were observed, whereas no significant differences in the risks of MI and revascularization were identified (Fig. 1). The risk of MACE, MI, stroke, or revascularization in the endoscopic resection group showed no significant difference from that observed in the control group. In the subgroup analyses, no significant interactions were identified between the study groups and baseline variables (Supplementary Fig. 2, Supplemental Digital Content 1, http://links.lww.com/JS9/C305).

### Effect of unmeasured confounding

A protective association between gastrectomy and cardiovascular events has been consistently observed in most scenarios with unmeasured confounding (Table 3). Endoscopic resection showed no significant association with cardiovascular events in the presence of modest unmeasured confounding factors, whereas endoscopic resection increased MACE in the presence of strong unmeasured confounding factors. The *E*-values for non-null associations in the gastrectomy vs. control group were 2.47 for MACE, 2.36 for MI, 2.22 for stroke, and 3.65 for revascularization.

**Table 3:** Hazard Ratios for cardiovascular event from sensitivity analyses for unmeasured confounding variables.

		U is a modest risk factor (Risk ratio _UD_ = 1.5)		U is a strong risk factor (Risk ratio _UD_ = 3.0)		*E* value
Comparison	Primary analysis	Modest imbalance (P _U1_ = 0.5, P _U0_ = 0.6)	Large imbalance (P _U1_ = 0.5, P _U0_ = 0.75)	Modest imbalance (P _U1_ = 0.5, P _U0_ = 0.6)	Large imbalance (P _U1_ = 0.5, P _U0_ = 0.75)	Effect estimate (95% CI)
Gastrectomy group vs. control						
MACE	0.65 (0.61–0.69)	0.67 (0.63–0.72)	0.71 (0.67–0.76)	0.71 (0.67–0.76)	0.81 (0.76–0.86)	2.47 (2.26)
MI	0.67 (0.59–0.76)	0.69 (0.61–0.79)	0.73 (0.64–0.84)	0.73 (0.64–0.84)	0.84 (0.73–0.96)	2.36 (1.94)
Stroke	0.70 (0.64–0.76)	0.73 (0.67–0.79)	0.77 (0.71–0.84)	0.77 (0.71–0.84)	0.87 (0.80–0.95)	2.22 (1.96)
Revascularization	0.47 (0.42–0.53)	0.49 (0.44–0.56)	0.52 (0.46–0.59)	0.52 (0.46–0.59)	0.59 (0.53–0.67)	3.65 (3.15)
Endoscopic resection group vs. control						
MACE	1.07 (0.91–1.26)	1.11 (0.95–1.31)	1.18 (1.00–1.39)	1.18 (1.00–1.39)	1.34 (1.14–1.58)	NA
MI	1.10 (0.78–1.54)	1.14 (0.81–1.60)	1.21 (0.86–1.70)	1.21 (0.86–1.70)	1.37 (0.98–1.93)	NA
Stroke	1.10 (0.88–1.37)	1.14 (0.92–1.42)	1.21 (0.97–1.50)	1.21 (0.97–1.50)	1.37 (1.11–1.71)	NA
Revascularization	1.03 (0.78–1.36)	1.07 (0.81–1.41)	1.13 (0.86–1.49)	1.13 (0.86–1.49)	1.29 (0.98–1.70)	NA

MACE, major adverse cardiovascular event; MI, myocardial infarction; NA, not assessable.

E indicates gastric cancer treatments (gastrectomy or endoscopic resection) exposure (E=1) vs. control (E=0), D indicates the composite event, and U indicates unmeasured confounder. P _U1_ indicates the prevalence of U in the patients with gastric cancer and P _U0_ indicates the prevalence of U in the control population.

**Figure 3:**
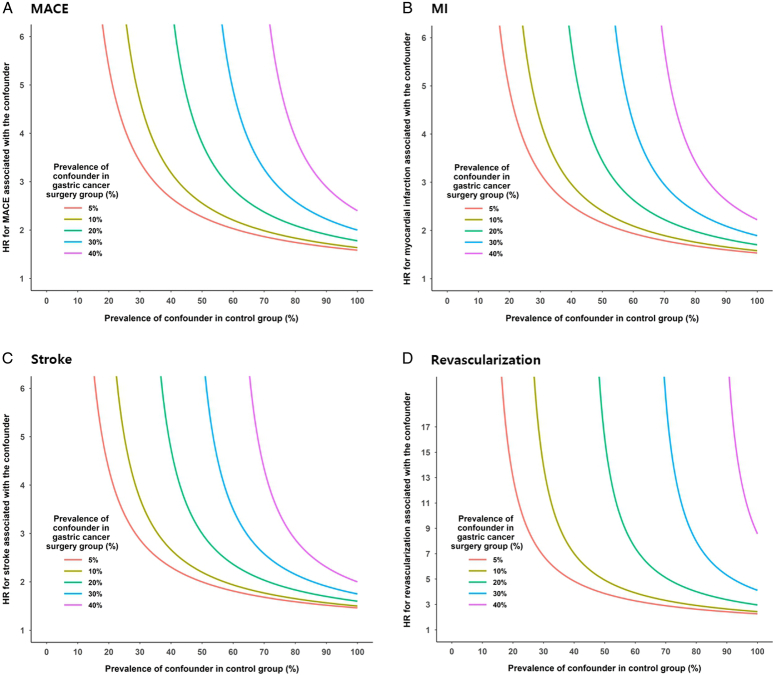
Effect of unmeasured confounding factors (gastrectomy vs. control population). Sensitivity analysis illustrates how powerful a single confounder would need to be to account for the cardiovascular protective effect of gastrectomy detected in the adjusted analysis. A single unmeasured confounder could produce the observed cardiovascular risk differences only if it increased the risk of (A) MACE, (B) MI, (C) stroke, and (D) revascularization, with HR of 2.2–3.7. MACE, major adverse cardiovascular event; MI, myocardial infarction; HR, hazard ratio.

Figure 3 shows the method used to determine whether an unmeasured binary risk factor can explain the HR of the overall results. The *x*-axis represents the hypothetical prevalence of unmeasured confounders in the control population, and the *y*-axis represents the hypothetical hazard ratio for cardiovascular events associated with this confounder. The curved lines indicate the hypothetical prevalence (5, 10, 20, 30, or 40%) of potential confounders in the gastrectomy group. A single unmeasured confounder could produce observed differences in cardiovascular risk only if it increased the risk of MACE, MI, stroke, and revascularization by an HR of 2.2–3.7.

## Discussion

Globally, South Korea has the highest 5-year survival rate (˃77% in the late 2010s) of patients with gastric cancer^4,5^. This could be attributed to the widespread implementation of gastric cancer screening through the National Cancer Screening Program. In the late 2010s, more than 63% of patients with gastric cancer in South Korea were detected early in the localized stage, according to the SEER staging system^6^. Therefore, South Korea has established an optimal patient population for the study of comorbidities in long-term surviving patients with gastric cancer. Considering the global trend of early detection and long-term survival in patients with gastric cancer, South Korea will serve as a reference for health predictions in other countries. The finding that cardiovascular risk is reduced after gastric cancer surgery is meaningful because it not only provides characteristic epidemiological data for patients with gastric cancer but also provides evidence for the hypothesis that gastric cancer surgery itself has metabolic effects.

Gastric cancer surgery alters cardiovascular risk factors. Body weight is an important predictor of cardiovascular risk, and patients with gastric cancer experience an average weight loss of 5–15% after surgery^7,8^. Weight loss in obese or overweight patients reduces cardiovascular risk^9^. Whether normal-weight patients with gastric cancer can also experience the cardiovascular benefits of weight loss after surgery remains questionable, and a study suggesting this possibility has recently been published. A national cohort study found that the incidence of type 2 diabetes was significantly decreased in normal-weight patients with gastric cancer who experienced weight loss after surgery^8^. This result suggests that normal-weight patients with gastric cancer may also experience the beneficial effects of weight loss after surgery. In addition, our results should be interpreted in the context of the specific situation in South Korea, where weight loss due to advanced-stage cachexia is less common than in other national cohorts. The possibility of bias due to unmeasured variables, such as stage or cachexia, was minimized because the analysis results, which considered unmeasured confounding factors, were consistent with the overall results (Table 3).

Gastric cancer surgery can alter the course of diseases such as type 2 diabetes^10,11^, hypertension^12^, and dyslipidemia^14^, which are potential risk factors for cardiovascular diseases. In particular, over 25% of patients with type 2 diabetes experience normoglycemia remission without diabetes medication within 3 years of gastric cancer surgery^11^. Patients with gastric cancer without type 2 diabetes before surgery have a reduced risk of developing type 2 diabetes by up to 35% after surgery^8^. Physiologically, patients who have undergone gastric cancer surgery experience systematic changes in glucose metabolism (e.g. increased small bowel glycolysis and increased white adipose tissue glucose uptake)^15^. An improvement in cardiovascular risk factors after gastric cancer surgery can be considered a cause of reduced cardiovascular risk after gastric cancer surgery.

An array of endocrine changes associated with gastric cancer surgery contributed to cardiovascular risk reduction. A previous study revealed that glucagon-like peptide 1 (GLP-1), known for its cardioprotective effects and modulation of endothelial dysfunction, is significantly increased after gastric cancer surgery^5^. Changes in adipokine levels, accompanied by postoperative weight loss primarily driven by the reduction of visceral adipose tissue, may also contribute to a reduction in cardiovascular risk^6^. Most notably, adiponectin, known for its anti-inflammatory and potent cardioprotective effects against ischemic/reperfusion injury, is markedly elevated after gastric cancer surgery^18–20^. In addition, previous studies have observed a decrease in postoperative leptin levels, a proinflammatory cytokine associated with leptin-associated hypertension^21,22^. Plasminogen activator inhibitor-1 (PAI-1), an inflammatory adipokine known for its association with dysregulation of the fibrinolytic pathway and thus MACE, is also decreased, likely due to the postoperative loss of visceral fat^18,23^.

To improve the reliability of the study results, we performed an unmeasured confounding analysis (Table 3). The NHIS database is limited in that it cannot obtain information on the stage of gastric cancer. However, it can use information on cardiovascular drugs, comorbid diseases, and lifestyle habits that can affect cardiovascular risk. Unmeasured confounding analysis could minimize the risk of residual bias due to unmeasured variables, thus changing the significance or size of the study results. This statistical method assumes the existence of unmeasured variables and calculates the HR by setting the incidence of these variables in various ways^16^. No evidence of bias due to unmeasured confounding factors was observed in our study.

This study had some limitations. First, although all available variables were utilized, owing to the retrospective design of the study, unavailable potential confounders might not have been adjusted. For instance, some cancer-related confounding factors may affect cardiovascular risk. As discussed above, we attempted to minimize the effects of unmeasured variables (e.g. chemotherapy or cachexia) by performing an unmeasured confounding analysis. Second, the limitations of using claims data, including possible coding errors, misdiagnoses, and misclassifications, must be considered. Third, concerns remain regarding reverse causation, although we washed out MACE within 2 years after surgery or endoscopic resection for cancer. Forth, our research design did not capture changes in cardiovascular risk factors after surgery. Further studies are needed to elucidate how changes in cardiovascular risk factors following surgery influence the observed reduction in cardiovascular risk after gastric cancer surgery.

In conclusion, our study was the first to show that patients with gastric cancer who have undergone gastrectomy have a reduced risk of cardiovascular disease compared to the general population. In contrast, patients with gastric cancer who underwent endoscopic resection did not show a significant difference in cardiovascular risk compared with the general population after treatment. Our results, in addition to their public health importance for the management of cardiovascular risk in patients with gastric cancer, will help patients and healthcare providers make more informed decisions regarding gastric cancer surgery. In addition, our results provide important research hypotheses regarding the metabolic effects of gastric cancer surgery, such as a reduction in cardiovascular risk.

## Ethical approval

This study was approved by the Institutional Review Board (Korea University Anam Hospital, no. 2019AN0156), which waived the requirement for informed consent because the customized database was released after de-identification and anonymization.

## Source of funding

This work was supported by the Basic Science Research Program through the National Research Foundation of Korea (grant no. 2020R1I1A1A01070106, for Y.K.), and a Korea University grant (for S.P.).

## Author contribution

Y.K., D.K., S.P., J.-W.K.: concept and design; Y.K. and S.K.: drafting of the manuscript; Y.K., D.K., S.P., and J.-W.K.: statistical analysis; S.P., J.-W.K.: administrative, technical, or material support; S.P. and J.-W.K.: supervision. All authors contributed in acquisition, analysis, or interpretation of data and critical revision of the manuscript for important intellectual content.

## Conflicts of interest disclosure

None reported.

## Research registration unique identifying number (UIN)

1. Name of the registry: The Research Registry.
2. Unique identifying number or registration ID: researchregistry9790.
3. Hyperlink to your specific registration (must be publicly accessible and will be checked): https://www.researchregistry.com/browse-the-registry#home/.

## Guarantor

Jin-Won Kwon, MPH, PhD, Professor, BK21 FOUR Community-Based Intelligent Novel Drug Discovery Education Unit, College of Pharmacy and Research Institute of Pharmaceutical Sciences, Kyungpook, National University, 80, Daehakro, Bukgu, Daegu 41566, Korea. Tel.: +82 53 950 8580; fax: +82 53 950 8557 E-mail: jwkwon@knu.ac.kr.

## Data availability statement

The underlying data from the electronically database is not available, as National Health Insurance Service in Korea provides data only if approved by the Institutional Review Board.

## Provenance and peer review

Not commissioned, externally peer-reviewed.

## Supplementary Material

SUPPLEMENTARY MATERIAL
