# Supplementary material for: Long-term cardiovascular risk reduction after gastric cancer surgery: a nationwide cohort study
Source: Int J Surg. 2024 Mar 27;110(7):4266–74. doi: 10.1097/JS9.0000000000001404 (PMC11254285; doi:10.1097/JS9.0000000000001404)

| **Supplemental Table 1.** Definition of comorbidities and cardiovascular medications | |
| --- | --- |
| Medical conditions | Definition, codes or conditions |
| Cancer | C code excluding C16 |
| Hypertension | (I10–I15) and ≥1 claim per year for hypertension medications |
| Diabetes | (E11-E14) and ≥1 claim per year for diabetes medications |
| Dyslipidemia | (E78) and ≥1 claim per year for dyslipidemia medications |
| Peripheral arterial disease | (I70.0, I70.1, I70.2, I70.8, I70.9, I73) |
| Heart failure | (I50) |
| Atrial fibrillation | (I48) |
| Transient ischemic attack | (G45) |
| Chronic kidney disease | (N18, N19) |
| Cardiovascular medications (available in South Korea) | |
| ACE inhibitors | captopril, enalapril, cilazapril, moexipril, perindopril, quinapril, ramipril, fosinopril |
| Angiotensin receptor blockers | telmisartan, valsartan, fimasartan, losartan, candesartan, irbesartan, eprosartan, olmesartan |
| Antiarrhythmics | flecainide, pilsicainide, propafenone, amiodarone, dronedarone, sotalol |
| Antiplatelets | abciximab, aspirin, limaprost, tirofiban, triflusal, dipyridamole, clopidogrel, prasugrel, ticagrelor |
| β-blockers | atenolol, bisoprolol, carvedilol, metoprol, nebivolol, propranolol, labetalol, nadolol, artinolo, betaxolol, bevantolol, esmolol, trandate |
| Calcium-channel blockers | amlodipine, felodipine, nicardipine, nifedipine, verapamil, diltiazem, benidipine, cilnidipine, efonidipine, isradipine, lacidipine, lercanidipine, nisoldipine |
| Diuretics | spironolactone, torsemide, chlorothiazide, hydrochlorothiazide, furosemide |
| Statins | lovastatin, simvastatin, Fluvastatin, pitavastatin, atorvastatin, rosuvastatin, pravastatin |
| Characters and numbers in parentheses are ICD-10-CM codes | |

**Supplemental Figure 1.** Flow diagram of the study population selection.

Abbreviations: MACE, major adverse cardiovascular event; MI, myocardial infarction


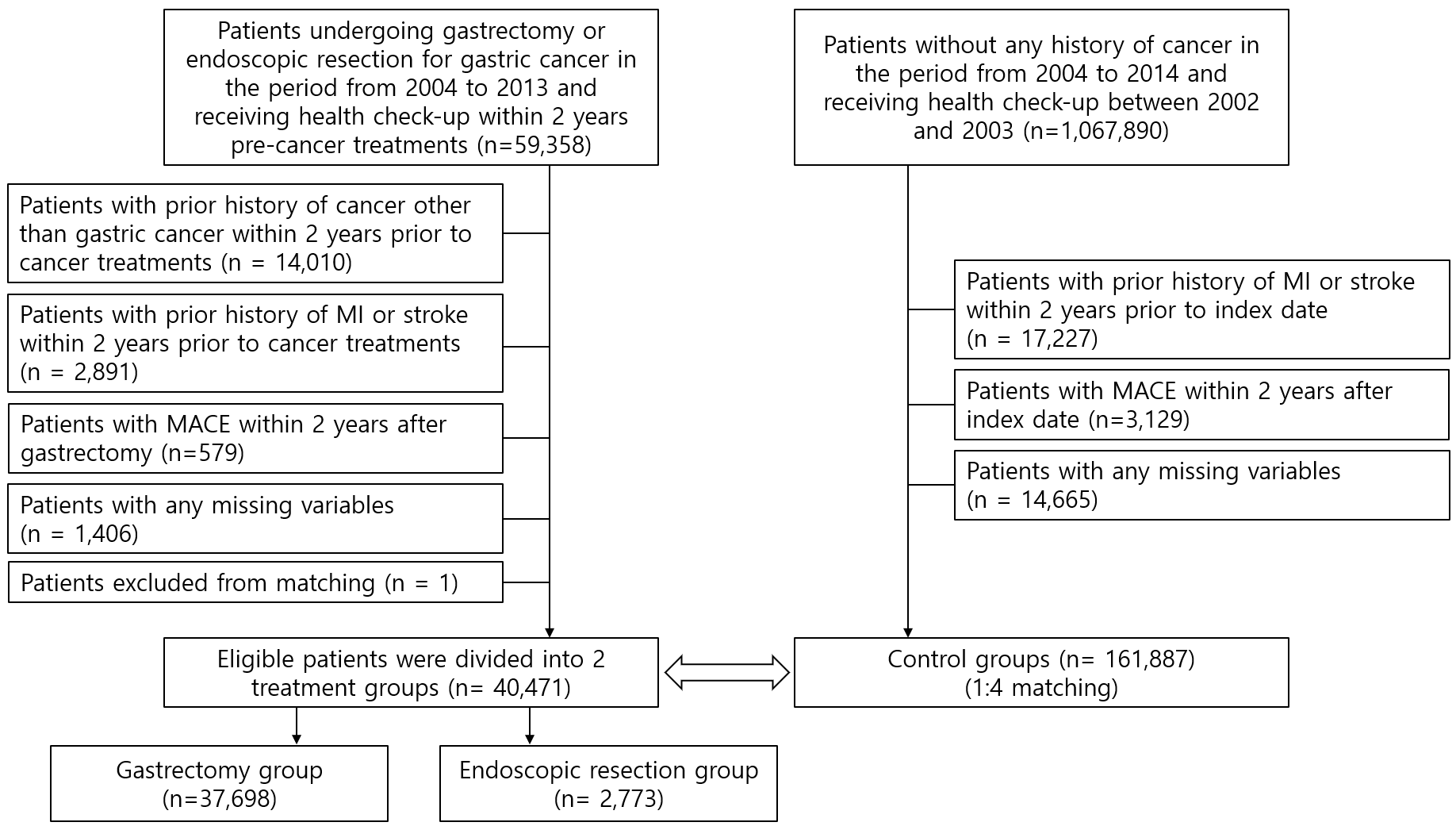


**Supplemental Figure 2**. Subgroup analyses according to baseline characteristics (endoscopic resection vs. control population): (A) MACE, (B) MI, (C) stroke, and (D) revascularization

Abbreviations: MACE, major adverse cardiovascular event; MI, myocardial infarction

(A) MACE


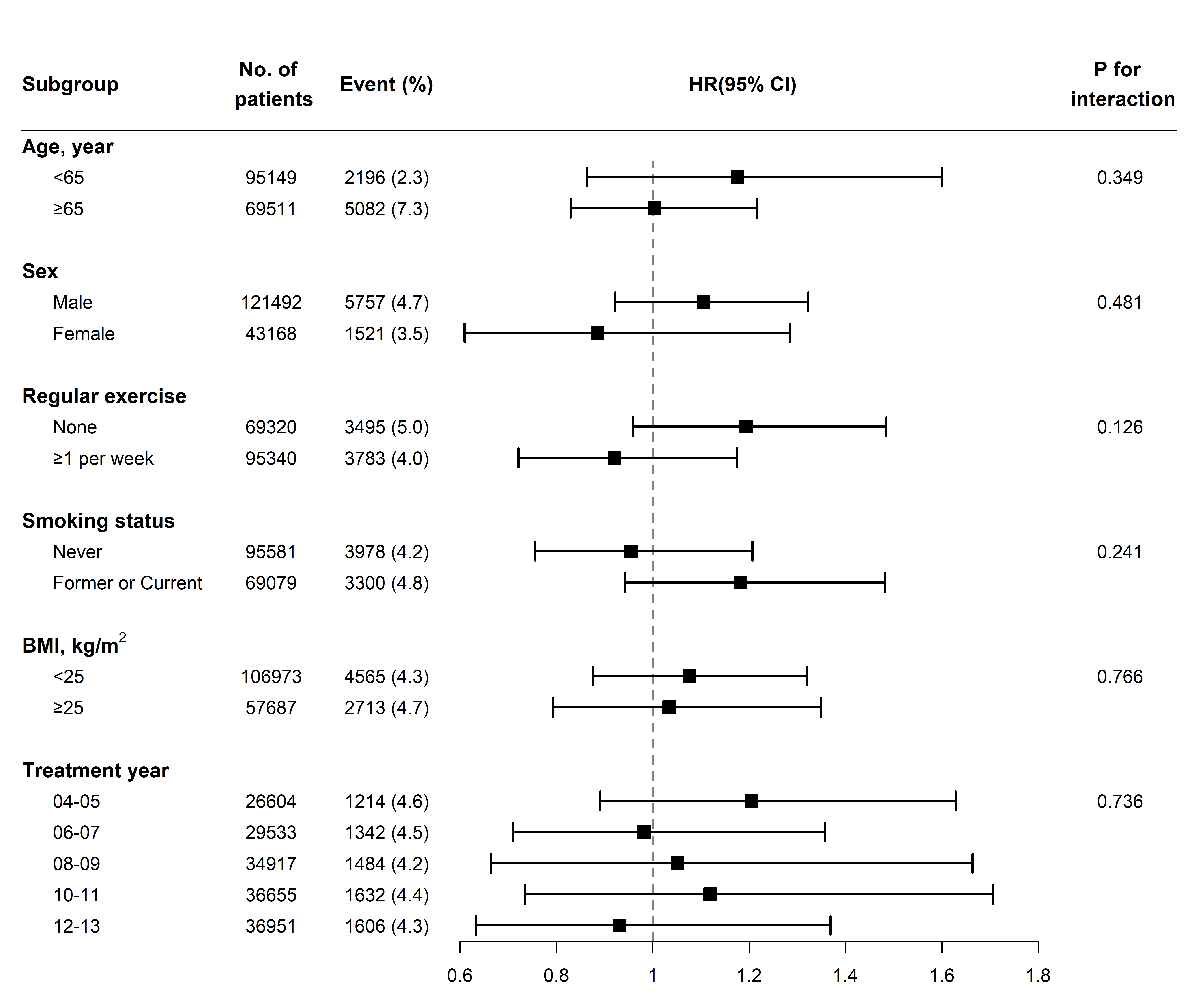


(B) MI


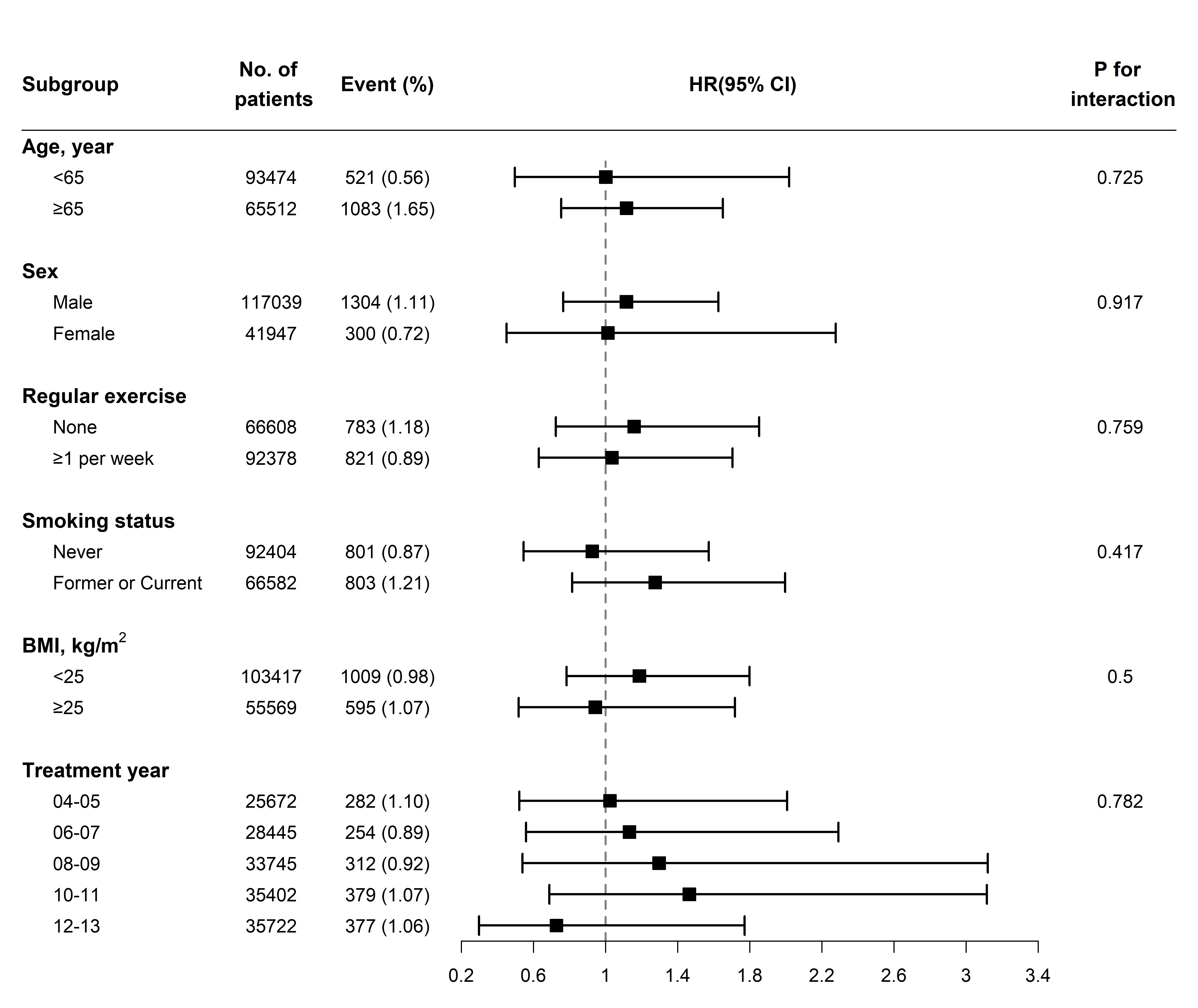


(C) Stroke


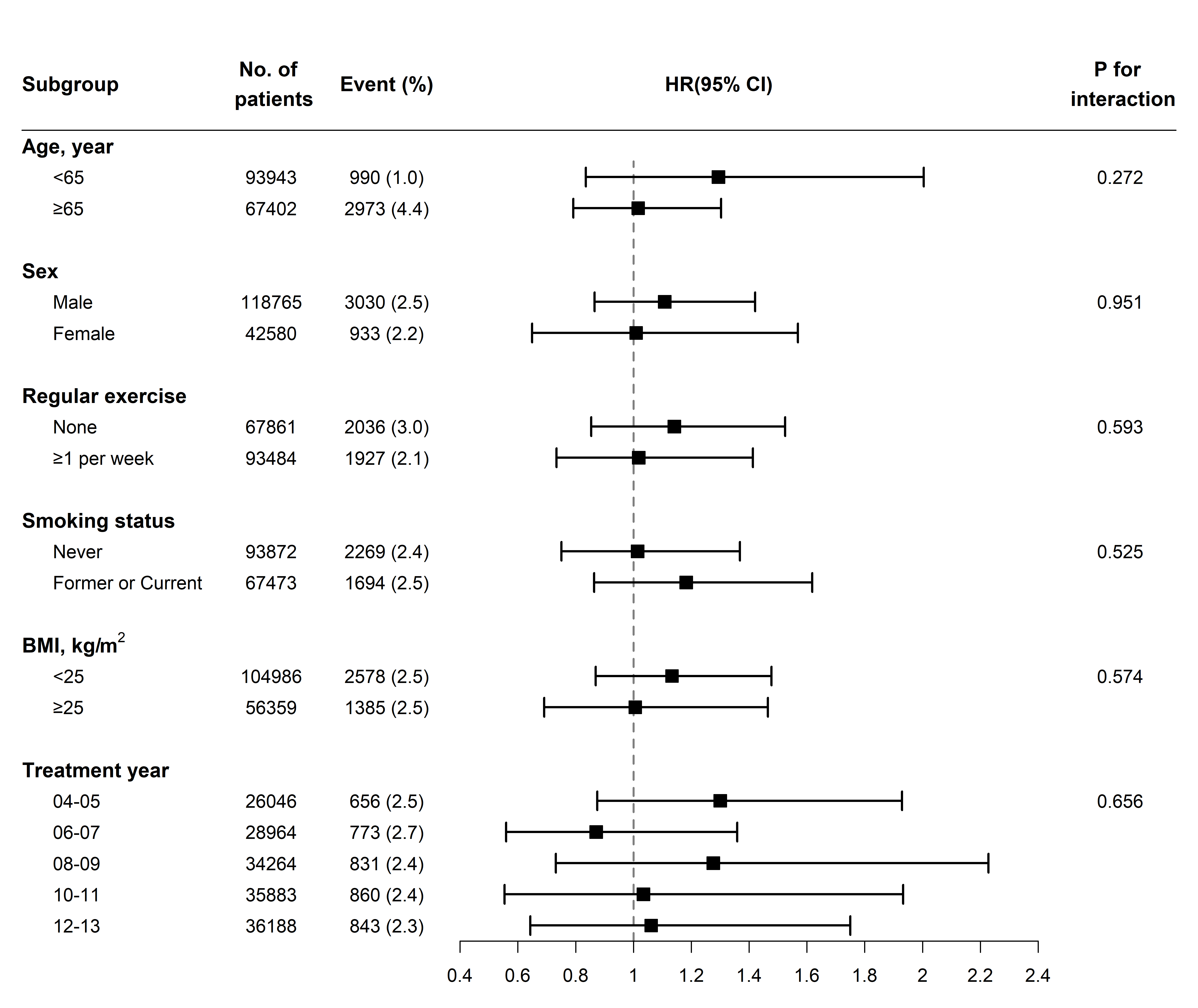


(D) Revascularization


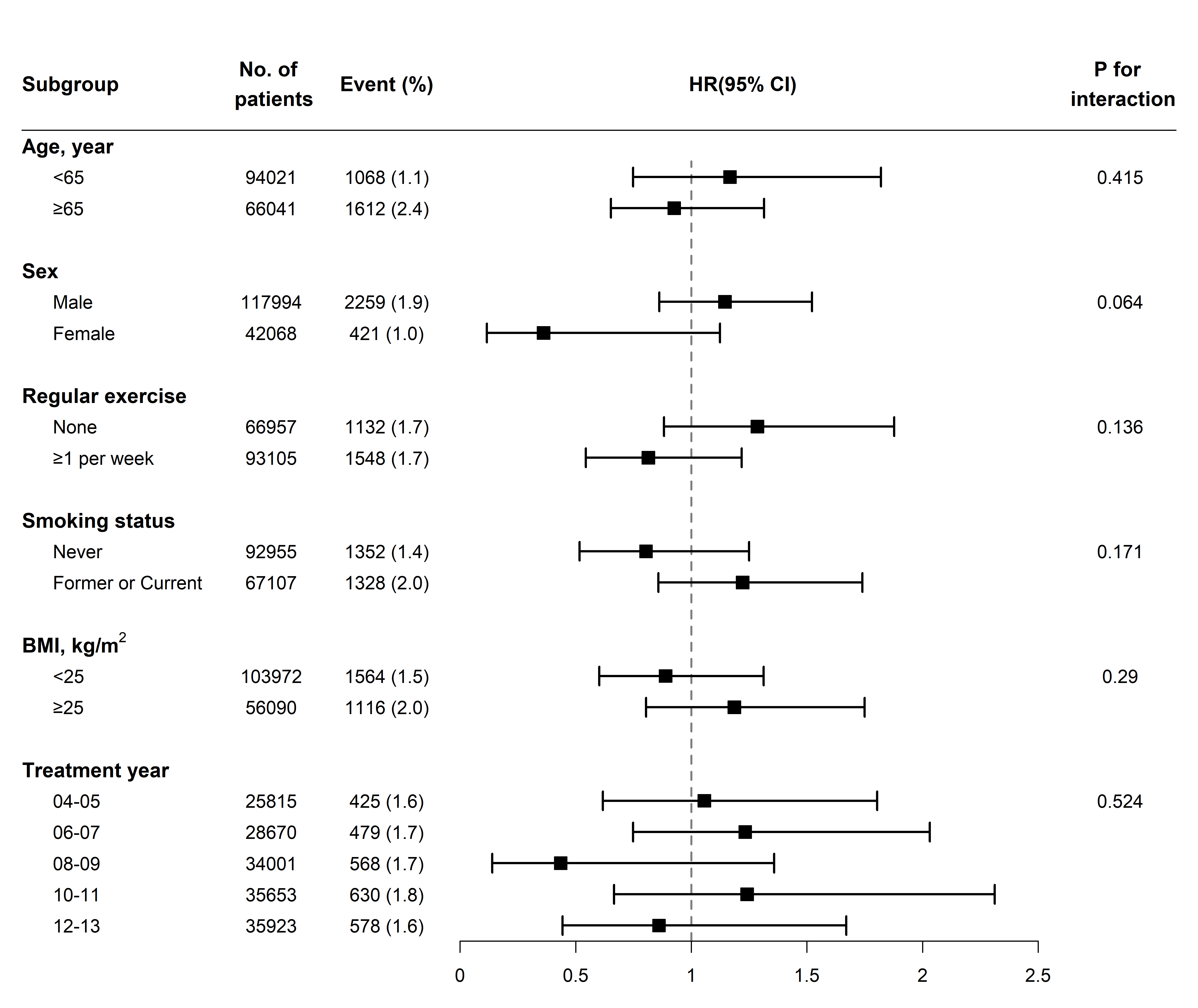

Supplement: SUPPLEMENTARY MATERIAL [file js9-110-4266-s001.docx]
